# The Effects of a Pre-Extubation Single Recruitment Maneuver on Ultrasonographic Lung Conditions in Patients Undergoing Lateral Decubitus Surgery: A Randomized Clinical Trial

**DOI:** 10.3390/jcm14092969

**Published:** 2025-04-25

**Authors:** Emre Sertaç Bingül, Meltem Savran Karadeniz, Mert Canbaz, Emre Şentürk, Cansu Uzuntürk, Selçuk Erdem, Nüzhet M. Şentürk

**Affiliations:** 1 Department of Anaesthesiology and Reanimation, Istanbul Faculty of Medicine, Istanbul University, 34093 Istanbul, Turkey; emre.bingul@istanbul.edu.tr (E.S.B.); mcanbaz@ku.edu.tr (M.C.);; 2 Department of Anaesthesiology and Reanimation, Acıbadem Fulya Hospital, 34349 Istanbul, Turkey; 3 Division of Urologic Oncology, Department of Urology, Istanbul Faculty of Medicine, Istanbul University, 34093 Istanbul, Turkey; 4 Department of Anaesthesiology and Reanimation, School of Medicine, Acıbadem University, 34752 Istanbul, Turkey

**Keywords:** lung ultrasound, postoperative pulmonary complications, lung injury, atelectasis, lung ultrasound score

## Abstract

**Background**: Upper abdominal surgeries exceeding two hours and operated in a lateral decubitus position present an “intermediate” risk for pulmonary complications. The objectives of this study were to observe the sonographic and clinical changes during and after surgeries with one recruitment maneuver (RM) performed intraoperatively before extubation. **Methods**: Laparoscopic nephrectomy patients were randomized into pre-extubation single RM (Group RM) and control (Group NoRM) groups. The LUS (Lung Ultrasound Score) was evaluated after intubation (T1), at the end of surgery before the RM (T2), after the RM but before extubation (T3), and 30 min after arrival to the Post-Anesthesia Care Unit (T4) in Group RM; in Group NoRM, it was evaluated at the T1, T2, and T4 time points. The primary outcome was the effect on the pre-extubation LUS (T2 in Group NoRM versus T3 in Group RM). The secondary outcomes included the effects on the T4 LUS, PPC occurrence, and PaO_2_/FiO_2_ ratios, and the sensitivity and specificity of the LUS in predicting PPCs. **Results**: The data of 54 patients were analyzed. The pre-extubation LUS was significantly lower in Group RM (16 (12.5, 17) vs. 18 (17, 20), *p* < 0.001). The T4 LUS was only different in the upper zones in the dependent lung (2 (1, 3.5) in Group RM vs. 4 (3, 4.5) in Group NoRM, *p* = 0.01). The perioperative PaO_2_/FiO_2_ ratios were similar (*p* > 0.05). The pre-extubation LUS exhibited 91% sensitivity (*p* = 0.04), whereas the T4 LUS sensitivity was 82% (*p* = 0.01). The PPC risk was 10-fold higher in patients with a pre-extubation LUS exceeding 19. **Conclusions**: A pre-extubation single RM instantly increases the LUS. However, this does not persist postoperatively or diminish respiratory complications. More importantly, the LUS was found to be a sensitive tool for predicting PPCs when performed just before extubation.

## 1. Introduction

The surgical position is a major determinant in the physical and physiological condition of the lungs during surgery [1]. A specific patient position is chosen in critical care settings to improve their functional residual capacity and oxygenation. However, it may become a matter of concern in patients with healthy lungs if they are operated in positions such as the lateral decubitus or prone position, since these positions may lower the compliance and ventilation volumes [2,3,4]. Considering the detrimental effects of general anesthesia, the eventual development of atelectasis due to such alterations may contribute to the occurrence of postoperative pulmonary complications (PPCs) [5,6].

With the integration of ultrasonography into clinical practice, physicians are now able to detect organ pathologies more rapidly and precisely. Lung ultrasound is widely used in critical care practice [7,8,9], and it is becoming more popular in anesthesiology practice. The Lung Ultrasound Score (LUS) has been defined as a pragmatic approach for quantitatively describing the lungs’ condition based on the A-lines (transverse lines reflecting normally aerated areas) or B-lines (longitudinal lines reflecting the loss of aeration to some extent) [9,10]. Accordingly, the two hemithoraxes are divided into six zones, which are each scored depending on the number or coalescence of pathologic B-lines. A higher LUS, which may reach up to a maximum of 36 points, indicates a worse lung condition with increased consolidation and aeration loss. Recruitment maneuvers are applied intraoperatively to improve oxygenation and are one way to reduce atelectasis formation.

In the lateral decubitus position, the lungs are named based on the amount of perfusion they receive, since perfusion is increased in the lower lung (“dependent lung”) due to gravity, whereas the upper lung is considered “non-dependent” [1]. This physiological property is considered “life-saving” because it provides more equitable ventilation/perfusion during thoracic surgery [11]. However, the case would be different in a completely healthy set of lungs since the distribution of aeration would be lower in the dependent lung. Laparoscopic nephrectomy appears to be a well-suited surgery for evaluating the effects of lateral positioning and general anesthesia on the lungs. This study aimed to determine whether a “single” recruitment maneuver before extubation can enhance the lung condition perioperatively, with the intention to examine the effects on PPC occurrence. Therefore, the primary outcome was the effect on the LUS before extubation. The secondary outcomes included the effects on the acute postoperative LUS, intra-group changes in the LUS, and the relationships between the LUS and oxygenation level and between the end-surgery and postoperative LUS and PPC occurrence.

## 2. Materials and Methods

Once approval from the local ethics committee was obtained (Istanbul University Istanbul Faculty of Medicine Clinical Research Ethics Committee-2022/1066), this trial was registered at clinicaltrials.gov (NCT05494255). Adult (>18 years of age) American Society of Anesthesiologists Physical Status I to III patients who were scheduled for an elective or semi-elective laparoscopic nephrectomy surgery and provided informed consent were enrolled in the study. Patients with existing lung disease, heart failure, risk of pneumothorax, and patients who required multiple intraoperative recruitment maneuvers, liberal fluid therapy (more than 10 mL/kg/h), or liberal blood product (more than 4 packs of erythrocyte suspension or fresh frozen plasma) replacement were excluded from the study. No imputation methods were applied to address missing data throughout the study. Therefore, the data of the excluded patients were not used in the statistical analyses.

### 2.1. Study Design

The current study was a single-center randomized controlled trial, and the patients were enrolled between August 2022 and May 2023. A simple randomization was performed using sealed opaque envelopes containing numbers indicating one of the groups, which were generated using a web-based number generator (www.sealedenvelope.com, seed no. 197561830999037). The envelopes were opened on the morning of the surgeries. According to the randomization, the patients were divided into intervention (Group RM) and control (Group NoRM) groups, and the intervention group received one recruitment maneuver following the completion of surgery, before extubation. The LUS evaluation was performed at several intraoperative time points. In order to reduce the probability of bias, the ultrasonographic assessments and scoring were performed based on the consensus of two anesthesiologists (MSK and ESB).

### 2.2. Perioperative Anesthesiologic Care of the Patients

Once the patient was admitted to the operating room, standard monitorization with electrocardiogram, pulse oximetry, and noninvasive blood pressure was provided. General anesthesia was induced intravenously (IV) via administering midazolam (2 mL/kg), propofol (2–5 mL/kg), fentanyl (3 mcg/kg), and rocuronium (0.6 mg/kg). After positioning the patient in the lateral decubitus position, invasive blood pressure monitoring from the radial artery was established, and anesthesia was continued with sevoflurane inhalation (with a MAC value of 1–1.2) and intravenous remifentanil infusion (0.1–0.2 mcg/kg). During the surgery, mechanical ventilation (MV) was set to 5 mmHg of the positive end-expiratory pressure (PEEP), at a frequency of 12–16 breaths/min and 6–8 mL/kg of tidal volume in the volume control mode. The intraoperative fluid replacement was kept between 4 and 8 mL/kg/h of crystalloids. Blood transfusion was not applied unless the hemoglobin levels dropped below 8 g/dL. Blood gas analyses were obtained in the 1st and 2nd hour of the operation, and 30 min after extubation in the Post-Anesthesia Care Unit (PACU). After the blood gas sampling in the PACU, the patients were taken into room air for fifteen minutes in order to observe if their SpO_2_ dropped. Patients with a SpO_2_ lower than 95% were considered room air test-positive.

### 2.3. Recruitment Maneuver and Lung Ultrasound Score (LUS) Assessment

**Figure 1:**
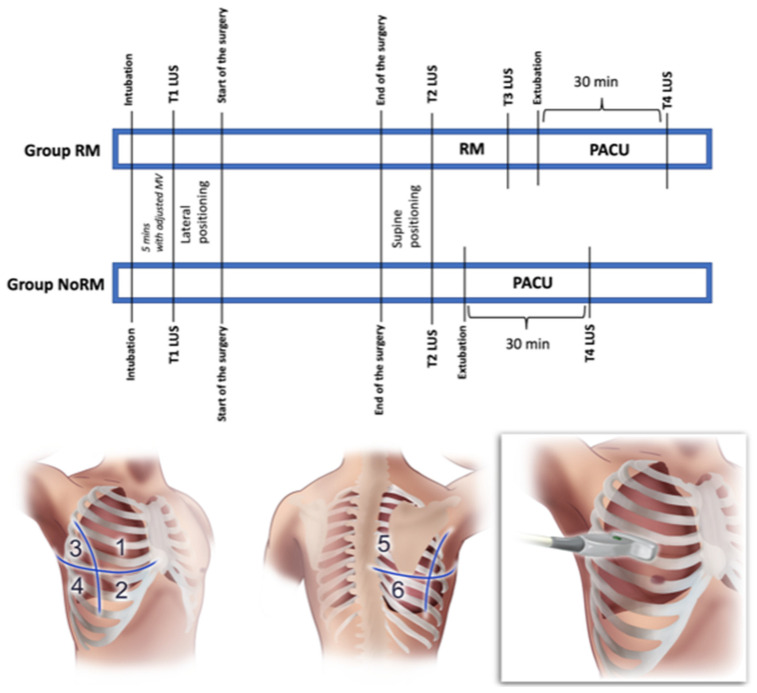
Schematics of intraoperative follow-up and transversal scanning lung ultrasound for six different lung zones. RM: recruitment maneuver; LUS: Lung Ultrasound Score; PACU: Post-Anesthesia Care Unit.

All of the ultrasound assessments were made in the supine position. In Group RM, the LUS was evaluated after intubation with the routine MV settings (T1), after the end of surgery (T2), after the RM (which was performed right after the T2 LUS assessment) (T3), and 30 min after the extubation (T4). Since the recruitment maneuver was not performed in Group NoRM, the LUS evaluation time points were defined as after intubation (T1), end of surgery (T2), and 30 min after the extubation (T4). Only one RM was performed in Group RM, and if more than one RM was required due to hypoxia, the patient was excluded from the study. In order to standardize the RM for the patients, the mechanical ventilation was set as follows: three cycles of breaths with an I/E ratio of 1:1, a respiratory rate of 6 breaths/min, and 35 cm H_2_O of peak pressure in the pressure control mode (Avance S5, GE Healthcare, Chicago, IL, USA) [12]. A brief description of the perioperative data collection timetable is presented in Figure 1.

A transversal scan of the lungs using a convex ultrasound probe (2–5 MHz, GE LOGIQ^™^, Milwaukee, WI, USA) was preferred for the lung ultrasound scoring. The probe was placed parallel to the ribs in the intercostal space at 6 separate quadrants in the two hemithoraxes, which were divided into “upper” and “lower” segments by a virtual line at the nipple level (Figure 1). According to the acquired image, every zone was given a score between 0 and 3 (0 points: A-lines reflecting completely normal aeration; 1 point: less than 4 B-lines reflecting mild loss of aeration; 2 points: more than 3 or coalescent B-lines reflecting moderate to severe loss of aeration; and 3 points: condensed lung areas with a distinct separation of the pleural sheets).

### 2.4. Outcome Measures

The primary outcome was assessed by comparing the pre-extubation LUS data (T2 LUS in Group NoRM versus T3 LUS in Group RM). Secondarily, the T4 LUS, incidence of a PPC, intraoperative and postoperative PaO_2_/FiO_2_ ratios, and incidence of a positive result in the room air test were compared between the groups to determine the effects of the pre-extubation single RM. Intra-group analyses were performed on the LUS at every time point. Apart from these pre-defined outcomes, post hoc analyses of whether the sensitivity and specificity of the pre-extubation LUS (T2 in Group NoRM and T3 in Group RM) and T4 LUS could predict postoperative pulmonary complication occurrence were conducted. As the patients were positioned in the lateral decubitus position for their nephrectomy surgery, the lungs were defined as dependent (inferior hemithorax) or non-dependent (superior hemithorax). The pulmonary complications were defined according to the EPCO classifications [13].

### 2.5. Statistical Analyses

Based on Park et al.’s study [14] and anticipating a 20% difference in the end-surgery LUS between the groups (LUS of 7.5 vs. 9.5), a group size of 25 patients was estimated (1:1 allocation) for a power of 95% and alpha error of 0.05. Considering a possible 20% drop-out rate, a total of 60 patients were enrolled in the study (G*Power 3.1).

The normality of the data distribution was tested using the Shapiro–Wilk test and histograms. Accordingly, Student’s *t*-test was used for homogenously distributed data, whereas heterogenous data were tested using the Mann–Whitney U-test. Categorical data were analyzed using the Chi-Square and Fisher exact tests if necessary. The results were expressed as the mean ± standard deviation for normally distributed data or median (25th percentile, 75th percentile) for skewed data. Non-parametric comparisons between groups were performed using the Wilcoxon Signed-Rank Test. When significant differences were detected, Bonferroni Correction was performed to adjust for multiple comparisons. Post hoc analyses were also performed to study the area under the curve (AUC) and the sensitivity–specificity Receiving Operator Curve (ROC). Multivariate logistic regression analysis was used for a risk analysis considering the cut-off LUS values. The results are given as the odds ratio and 95% confidence interval. The SPSS for Mac version 21 (SPSS Inc., Chicago, IL, USA) software package was used for the statistical analyses.

## 3. Results

**Figure 2:**
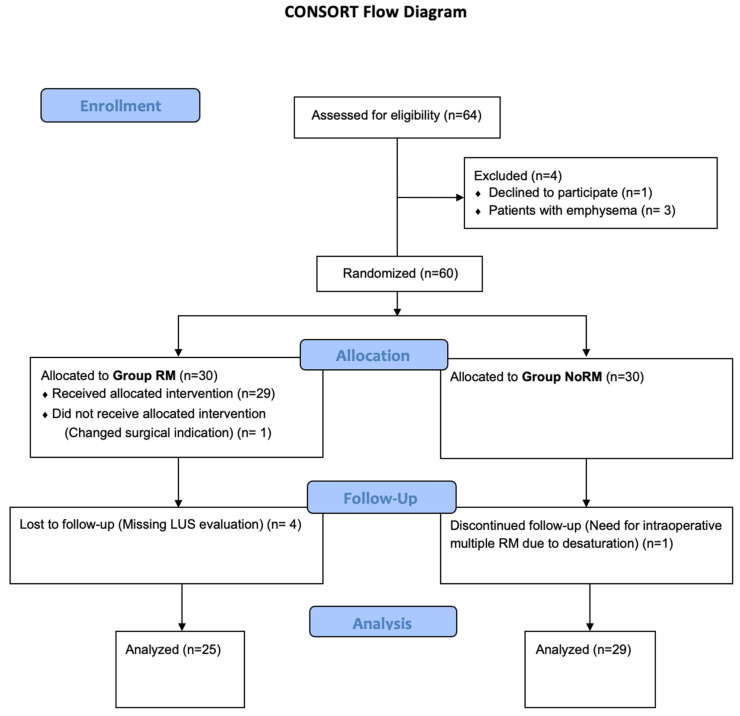
CONSORT diagram.

A total of 60 patients were randomly assigned to the two groups. In Group RM, one patient was excluded due to their surgery changing to an open procedure, and four patients were excluded due to missing intraoperative LUS evaluations. One patient was excluded from Group NoRM due to the need for multiple RMs. Accordingly, 25 patients in Group RM and 29 patients in Group NoRM were included in the final analyses. The CONSORT diagram is presented in Figure 2. The demographics of the two groups were similar, including postoperative pulmonary complications, room air test positivity in the PACU, surgery and anesthesia duration, ARISCAT scores, and total administered fluid parameters (Table 1).

**Figure 3:**
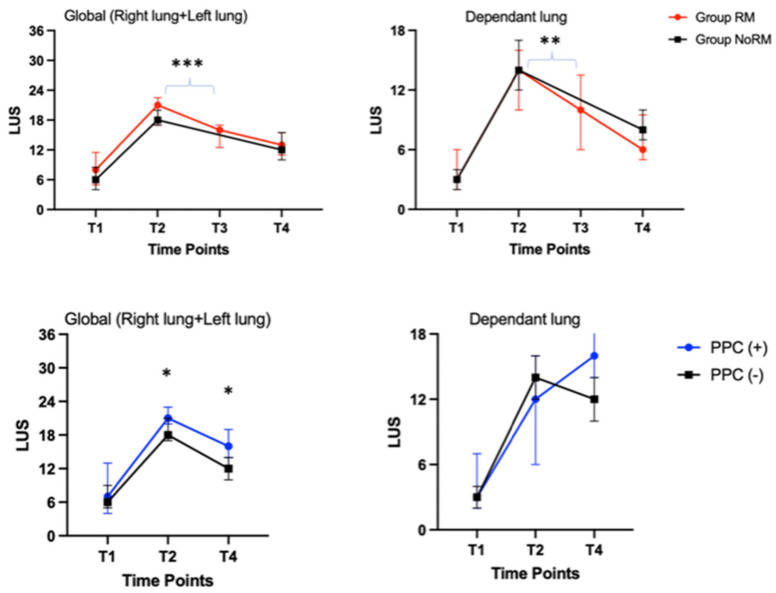
Both lungs and dependent lung median Lung Ultrasound Scores at different time points. LUS: Lung Ultrasound Score; RM: recruitment maneuver; PPC: postoperative pulmonary complication; T1: 5 min after intubation; T2: at the end of surgery before recruitment maneuver (in Group RM) or at the end of surgery before extubation; T3: after recruitment maneuver but before extubation; T4: 30 min after extubation in Post-Anesthesia Care Unit. * *p* < 0.05 and ** *p* = 0.002 (in comparison of T2 LUS in Group NoRM versus T3 LUS in Group RM); *** *p* < 0.001 (in comparison of T2 LUS in Group NoRM versus T3 LUS in Group RM).

The initial lung conditions (T1 LUS) of the patients were similar in both groups. The LUS before extubation was significantly lower in Group RM (16 (12.5, 17) vs. 18 (17, 20), *p* < 0.001). This difference was also observed when only evaluating the dependent lung (*p* < 0.05). However, the global lung assessments were similar between the groups in the PACU (13 (11, 15.5) in Group RM vs. 12 (10, 15.5) in Group NoRM, *p* = 0.4), except in the upper zones of the dependent lungs (2 (1, 3.5) in Group RM vs. 4 (3, 4.5) in Group NoRM, *p* = 0.01). The sonographic recovery of the lungs was validated via intra-group analyses of Group RM, which showed a lower LUS at T3 when compared to that at T2 (16 (12.5, 17) vs. 21 (17.5, 22), respectively; *p* < 0.001). When the patients were divided into two groups based on PPC occurrence, the end-surgery global (T2) and postoperative global (T4) LUSs were significantly higher in patients with PPCs (21 (20, 23) vs. 18 (17, 21), and 16 (14, 19) vs. 12 (10, 14), respectively; *p* < 0.05) (Table 2). The abovementioned data are summarized in Table 2 and Figure 3.

**Figure 4:**
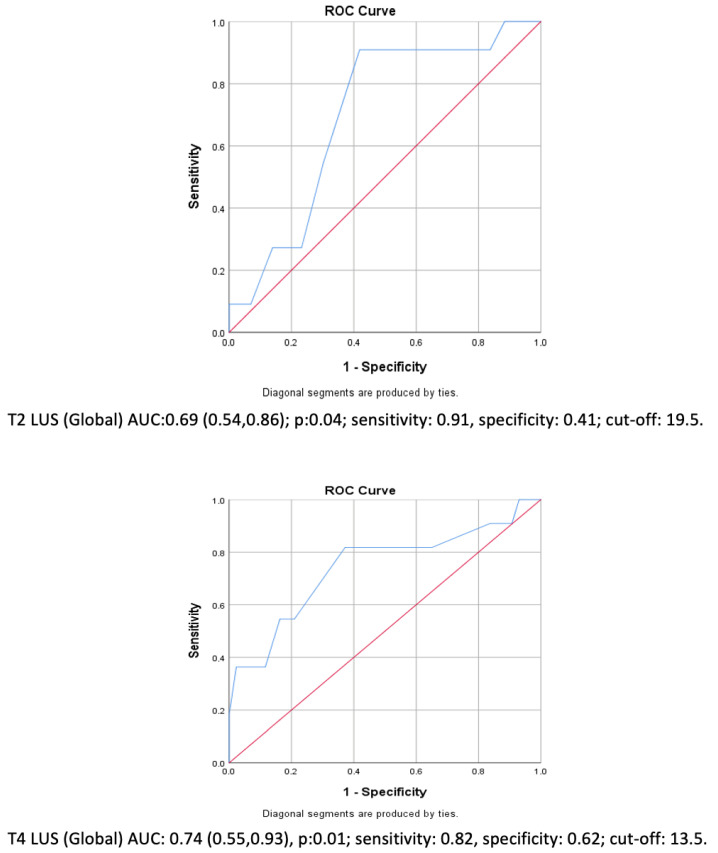
ROC analyses of pre-extubation and T4 LUS. Blue line shows the actual test in comparison to no predictive value (Red line).

On the other hand, the whole group’s pre-extubation global LUS (T2 in Group NoRM and T3 in Group RM) ROC analyses exhibited 91% sensitivity for predicting PPCs (area under the curve: 0.69 (0.54, 0.86); cut-off LUS: 19.5 (*p* = 0.04)). Similarly, the postoperative (T4) global LUS evaluation could also predict PPCs with a sensitivity of 82% (area under the curve: 0.74 (0.55, 0.93); cut-off LUS: 13.5 (*p* = 0.01)) (Figure 4). If these cut-off values are used to separate the whole group, the patients with a pre-extubation LUS more than 19 would have a 10-fold higher risk for a PPC (OR: 10.2; 95% CI: (1.14, 91.2); *p* = 0.04). However, using a “postoperative” LUS cut-off value of 13 did not result in a significant difference (*p* = 0.06) (Table 2).

Considering the lower drop-out rate than initially anticipated, the actual power of the current study was found to be 96%.

## 4. Discussion

In this randomized controlled trial, it was observed that a pre-extubation single RM was capable of enhancing the sonographic condition of the lungs. However, this effect diminished during the postoperative course. On the other hand, the LUS appears to be a promising tool for predicting PPCs when evaluated before extubation and in the acute postoperative period, and could be implemented into clinical practice as a practical method for surgeries that are prone to respiratory complications.

Lung ultrasound has been widely investigated in the literature, with it generally exhibiting good reliability for identifying “aerated” or “injured” regions of the lungs, which can then be tested for clinical follow-up or used for timing extubation under critical care settings [7,8]. The accuracy of this tool is well established and can even surpass conventional X-rays in certain cases [15,16,17]. Despite the accuracy of lung ultrasound, the use of the LUS has not been implemented in “perioperative” clinical practice. Quantitative scoring of the sonographic view of lungs may allow physicians to obtain a better understanding of the severity of the existing pathology. However, the published meta-analyses focused on lung ultrasound for pneumonia, respiratory distress, or heart failure screening, which mostly occur in emergency departments or critical care units [17,18]. Lung ultrasound usage in anesthesiology practice still relies on limited literature.

Among the limited publications, the study by Genereux et al. compared protective ventilation with “intermittent” RMs versus zero end-expiratory pressure ventilation; they found a better intraoperative LUS in the protective set-up group but similar “postoperative” lung conditions [19]. This was also true when the RMs were performed under ultrasound guidance. Confirming the lung inflation by visualizing it did not change the outcomes in laparoscopic gynecological surgeries [14]. These findings align with those of the current study. Performing an RM (either intermittently or as a single event) appeared to enhance the intraoperative lung sonography. However, it did not correlate with a sonographic or clinical improvement in the acute postoperative period.

**Figure 5:**
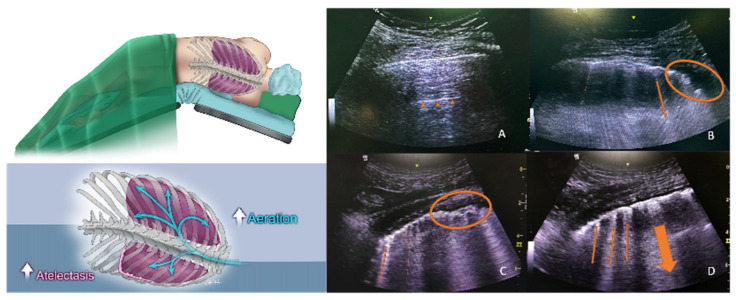
Diagrams and example images of pre-extubation lung ultrasound. (**A**) shows the non-dependent lung zone 4 with 0 points; small triangles indicate the physiologic A-line. (**B**–**D**) show the dependent lung zone 4 in different patients. The thin and dashed arrows indicate the B-lines. The thick arrows indicate converged B-lines, and circles indicate consolidation areas (3 points).

Monastesse et al. demonstrated a moderately correlated decrease in the PaO_2_/FiO_2_ ratio with a lower LUS in laparoscopic surgery patients who were operated on in the lithotomy position (r = −0.43) and, as expected, most LUS changes were observed in the inferior and posterior zones of the lungs [20]. Due to the surgical position, laparoscopic nephrectomy surgeries present a different perspective for respiratory investigations. Theoretically, with the lateral decubitus position, an increase in abdominal pressure and airway resistance is encountered, resulting in a decreased end-expiratory lung volume and static chest wall compliance in the “dependent” lung, which also receives the majority of the lung perfusion [21]. Therefore, consolidation and atelectasis are expected in the dependent lung during such surgeries, which may alter oxygenation, resulting in several pulmonary complications (Figure 5). Despite the abovementioned intraoperative changes in the dependent lung, the LUS of the dependent lungs cannot be considered as a major predictor of PPCs. Interestingly, only global (both lungs) LUS measurements showed significant changes when the groups were divided according to the presence of a PPC. The ultrasound scores for the upper and lower zones of the dependent lung did not show a statistically significant difference. Since our patients were operated on in the lateral position, one can assume that the condition of the “non-dependent” lung would be more clinically important.

The ARISCAT score is the most used tool for predicting PPCs, which has found that “upper abdominal surgeries” are an independent risk factor, with an odds ratio of 4.4. In the current study, the patient population demonstrated an “intermediate” risk, with an PPC incidence of 20%. Similarly, the PPC incidence in Canet et al.’s work was 17.7% for the same risk group [22]. Fu et al. compared protective ventilation (6–8 cmH_2_O PEEP and 6–8 mL/kg tidal volume) versus an 8–10 mL/kg tidal volume with zero end-expiratory pressure, and found a drop in the PPC incidence from 30% to 10%, which was consistent with the postoperative intermittent LUS examination results [23]. Despite using a similar ventilation modality, we did not observe such a low incidence rate. However, the LUS appears to be a valuable tool for predicting patients who are prone to developing pulmonary complications after surgery. We found that an “intraoperative” LUS exceeding 19 points right before extubation reflects a 91% sensitivity, indicating that a careful approach to the patient is needed. This proposition is validated by Szabo et al.’s research, which found a 94% sensitivity for the 24 h postoperative LUS [24]. However, 24 h after surgery could be considered within the “postoperative” period, and thus this assessment is a real-time diagnosis rather than a prediction. It should be noted that our study was not initially designed to determine if a high LUSis a risk factor for PPC development. Therefore, further clinical trials are needed to test if the LUS can predict PPCs. Nevertheless, ultrasound is an easy-to-perform bedside technique, which can be implemented in regular anesthesiology practice. Considering that the strongest PPC predictor is the “preoperative” ARISCAT score, with an 87% sensitivity for the “intermediate” risk group, ultrasound may offer a strong alternative for real-time on-site observations [22]. There is clear evidence showing the superiority of ultrasound for detecting “clinically relevant” pulmonary complications compared to chest X-rays in “perioperative” settings [25].

The subjectivity of the LUS is the major limitation of this study. To mitigate this issue, we had two experienced physicians come to a consensus on the LUS for every case; however, the lack of blinding of these physicians to the intervention represents a potential source of bias. Another limitation would be the lack of another patient group, for example, a group receiving multiple recruitment maneuvers. The current design was not able to exhibit a reduction in PPCs with a single RM; performing multiple RMs could provide a better insight. Yet, our sample size calculation was not built upon the hypothesis of a reduction in PPCs. Lastly, an intention-to-treat analysis for excluded patients would have given a more clarified understanding for interpretation of the dataset. Nevertheless, this does not invalidate the results of our study.

## 5. Conclusions

Performing a single recruitment maneuver right before the extubation instantly increases the global aeration of the lungs, as indicated by the LUS, and this effect diminishes in the acute postoperative period. Patients experiencing PPCs after laparoscopic nephrectomy surgery had a higher intra- and postoperative LUS.

The most important outcome of the current study is that scoring lungs via ultrasound is a sensitive tool in predicting PPCs, and physicians should be encouraged to use this practical tool in “perioperative” settings.

## Figures and Tables

**Table 1:** Demographic and perioperative data of the patients. Data are presented as *n* and percentage or median (Q1, Q3).

	Group RM (+) (*n* = 25)	Group NoRM (–) (*n* = 29)	*p*
Age (years)	52 (48, 57)	57 (51, 64.5)	0.3 ^a^
Gender			0.2 ^b^
Female	14, 56%	11, 38%	
Male	11, 44%	18, 62%	
BMI (kg/m^2^)	29 (26.5, 29)	27 (24, 29.5)	0.1 ^a^
BMI > 30 (n, %)	5, 20%	7, 24%	0.7 ^b^
ASA-PS			0.06 ^b^
ASA I	11, 44%	5, 17%	
ASA II	13, 52%	19, 66%	
ASA III	1, 4%	5, 17%	
Functional capacity			0.1 ^c^
I	24, 96%	23, 79%	
II	1, 4%	6, 21%	
ARISCAT score	38 (38, 41)	41 (34, 41)	0.8 ^a^
Comorbidities			
Hypertension	10, 40%	14, 48%	0.5 ^b^
Diabetes mellitus	4, 16%	4, 19%	1 ^c^
Chronic obstructive pulmonary disease	1, 4%	1, 3%	1 ^c^
Smoking	10, 40%	9, 31%	0.5 ^b^
Ischemic heart disease	1, 4%	3, 14%	0.4 ^c^
Renal failure	0, 0%	2, 7%	0.5 ^c^
Endocrinologic disease	1, 4%	4, 14%	0.4 ^c^
Postoperative Pulmonary Complications	6, 24%	5, 17%	0.5 ^b^
New requirement for O_2_ or respiratory support	0	1, 3%	
Aspiration pneumonitis	0	0	
Pneumonia	2, 4%	2, 6%	
ARDS	0	0	
Pneumothorax	0	0	
Atelectasis	4, 8%	2, 6%	
Bronchospasm	0	0	
PACU room air test (+)	6, 24%	11, 38%	0.3 ^b^
Surgery duration (min)	215 (175, 275)	220 (190, 260)	0.8 ^a^
Anesthesia duration (min)	240 (210, 300)	240 (225, 300)	0.7 ^a^
Total fluid administered intraoperatively (mL)	2500 (2000, 3300)	2500 (1800, 3100)	0.4 ^a^
Intraoperative first hour PaO_2_/FiO_2_	255 (245, 320)	266 (264, 362)	0.1 ^a^
PACU PaO_2_/FiO_2_	416 (410, 480)	296 (245, 412)	0.5 ^a^

^a^: Mann–Whitney U-test, ^b^: Chi-Square test, ^c^: Fisher’s exact test. Q1: 25th percentile; Q3: 75th percentile; RM: recruitment maneuver; ARDS: Acute Respiratory Distress Syndrome; ASA-PS: American Society of Anesthesiologists Physical Status; FC: functional capacity; ARISCAT: Assessment of Respiratory Risk in Surgical Patients in Catalonia; PACU: Post-Anesthesia Care Unit.

**Table 2:** Lung Ultrasound Score data. Data are presented as the median (Q1, Q3).

Lung Ultrasound Score Assessment (LUS)	Group RM	Group NoRM	*p*
T1 LUS			
Dependent lung lower zones (zones 2+4+6)	3 (2, 4)	2 (1, 3)	0.1
Dependent lung upper zones (zones 1+3+5)	1 (0, 2)	1 (0, 2)	0.8
Dependent lung (total)	3 (2, 6)	3 (2, 4)	0.2
Global (right lung + left lung)	8 (5, 11.5)	6 (4, 8.5)	0.2
T2 LUS			
Dependent lung lower zones (zones 2+4+6)	8 (6, 8)	8 (7, 8)	0.8
Dependent lung upper zones (zones 1+3+5)	7 (4, 8)	6 (5, 8)	0.4
Dependent lung (Total)	14 (10, 16)	14 (12, 17)	0.5
Global (right lung + left lung)	21 (17, 22.5)	18 (17, 20)	0.09
Pre-extubation LUS measurements (T3 in Group RM vs. T2 in Group NoRM)			
Dependent lung lower zones (zones 2+4+6)	6 (4, 7)	8 (7, 8)	0.003 *
Dependent lung upper zones (zones 1+3+5)	4 (2, 6)	6 (5, 8)	0.002 *
Dependent lung (total)	10 (6, 13.5)	14 (12, 17)	0.002 *
Global (right lung + left lung)	16 (12.5, 17)	18 (17, 20)	<0.001 *
T4 LUS			
Dependent lung lower zones (zones 2+4+6)	4 (4, 5.5)	5 (3, 6)	0.8
Dependent lung upper zones (zones 1+3+5)	2 (1, 3.5)	4 (3, 4.5)	0.01 *
Dependent lung (total)	6 (5, 9.5)	8 (7, 10)	0.1
Global (right lung + left lung)	13 (11, 15.5)	12 (10, 15.5)	0.4
**Lung Ultrasound Score assessment (LUS)**	**PPC (+)**	**PPC (-)**	***p***
T1 LUS			
Dependent lung lower zones (zones 2+4+6)	3 (2, 4)	2 (1, 3)	0.1
Dependent lung upper zones (zones 1+3+5)	1 (0, 3)	1 (0, 2)	0.8
Dependent lung (total)	3 (2, 7)	3 (2, 4)	0.4
Global (right lung + left lung)	7 (4, 13)	6 (5, 9)	0.7
T2 LUS (in both groups)			
Dependent lung lower zones (zones 2+4+6)	7 (5, 8)	7 (5, 8)	0.3
Dependent lung upper zones (zones 1+3+5)	5 (1, 8)	7 (5, 8)	0.3
Dependent lung (total)	12 (6, 16)	14 (12, 16)	0.3
Global (right lung + left lung)	21 (20, 23)	18 (17, 21)	0.04 *
T4 LUS			
Dependent lung lower zones (zones 2+4+6)	5 (4, 6)	4 (3, 5)	0.3
Dependent lung upper zones (zones 1+3+5)	3 (1, 6)	3 (2, 4)	0.7
Dependent lung (total)	8 (5, 12)	7 (6, 10)	0.4
Global (right lung + left lung)	16 (14, 19)	12 (10, 14)	0.01 *
**LUS change due to single recruitment maneuver** **in Group RM**	**T2**	**T3**	
Dependent lung lower zones (2+4+6)	8 (6, 8)	6 (4, 7)	<0.001 **
Dependent lung upper zones (1+3+5)	7 (4, 8)	4 (2, 6)	<0.001 **
Dependent lung (total)	14 (10, 16)	10 (6, 13.5)	<0.001 **
Global (right lung + left lung)	21 (17, 22.5)	16 (12.5, 17)	<0.001 **
**Whole-Study-Group Regression Analysis (PPC occurrence)**	**OR (95% CI)**		
T2 LUS (in Group NoRM) + T3 LUS (in Group RM) Global > 19	10.2 (1.14, 91.2)		0.04 ***
T4 LUS Global > 13	5.2 (0.93, 29.9)		0.06

Q1: 25th percentile; Q3: 75th percentile; RM: recruitment maneuver; PPC: postoperative pulmonary complication; * Mann–Whitney U-test. ** Wilcoxon test; *** regression analysis.

## Data Availability

The clinical data of this trial are available upon reasonable request to the corresponding author.

## Abbreviations

The following abbreviations are used in this manuscript:

RM	recruitment maneuver
LUS	Lung Ultrasound Score
PPC	postoperative pulmonary complication
PEEP	positive end-expiratory pressure
ARISCAT	Analysis of Risk Index for Surgical Comorbidities and Anesthetic Technique
MAC	minimum alveolar concentration
PACU	Post-Anesthesia Care Unit
ASA	American Society of Anesthesiologists Physical Status
IV	intravenous
